# Topics and Trends of Health Informatics Education Research: Scientometric Analysis

**DOI:** 10.2196/58165

**Published:** 2024-12-11

**Authors:** Qing Han

**Affiliations:** 1 School of Medical Technology and Information Engineering Zhejiang Chinese Medical University Hangzhou China

**Keywords:** health informatics education, scientometric analysis, structural topic model, health informatics, medical informatics, medical education

## Abstract

**Background:**

Academic and educational institutions are making significant contributions toward training health informatics professionals. As research in health informatics education (HIE) continues to grow, it is useful to have a clearer understanding of this research field.

**Objective:**

This study aims to comprehensively explore the research topics and trends of HIE from 2014 to 2023. Specifically, it aims to explore (1) the trends of annual articles, (2) the prolific countries/regions, institutions, and publication sources, (3) the scientific collaborations of countries/regions and institutions, and (4) the major research themes and their developmental tendencies.

**Methods:**

Using publications in Web of Science Core Collection, a scientometric analysis of 575 articles related to the field of HIE was conducted. The structural topic model was used to identify topics discussed in the literature and to reveal the topic structure and evolutionary trends of HIE research.

**Results:**

Research interest in HIE has clearly increased from 2014 to 2023, and is continually expanding. The United States was found to be the most prolific country in this field. Harvard University was found to be the leading institution with the highest publication productivity. Journal of Medical Internet Research, Journal of The American Medical Informatics Association, and Applied Clinical Informatics were the top 3 journals with the highest articles in this field. Countries/regions and institutions having higher levels of international collaboration were more impactful. Research on HIE could be modeled into 7 topics related to the following areas: clinical (130/575, 22.6%), mobile application (123/575, 21.4%), consumer (99/575, 17.2%), teaching (61/575, 10.6%), public health (56/575, 9.7%), discipline (55/575, 9.6%), and nursing (51/575, 8.9%). The results clearly indicate the unique foci for each year, depicting the process of development for health informatics research.

**Conclusions:**

This is believed to be the first scientometric analysis exploring the research topics and trends in HIE. This study provides useful insights and implications, and the findings could be used as a guide for HIE contributors.

## Introduction

Health informatics is at the crossroad of information science, computer science, data science, medicine, and health care, with great improvements in a wide range of health-related areas, including nursing, health care, public health, clinical decision, and biomedicine [1]. There is a growing need for professionals to be able to address health informatics issues in health-related fields through the design, development, implementation, evaluation, and use of innovative information technologies and applications.

Consequently, many academic and educational institutions are making significant contributions toward training health informatics professionals by offering educational programs or courses in the field [2]. The nature of health informatics attracts both health care and IT professionals as specialists in a highly interdisciplinary field. Health care professionals, on the other hand, need to grasp the opportunities and limitations that technology can bring to health care [3]. There are several ways to enter health informatics education (HIE) in different countries around the world, with the most common pathway being postgraduation following the completion of a bachelor’s degree in health informatics or a related degree in health care, life sciences, cognitive science, computer science and IT, health information management, and others, with associated work experience [4-10].

Due to its multidisciplinary foundations and diverse application areas, HIE has captured the interest of researchers from numerous areas. Engagement from these researchers has been beneficial to the field as it has led to the development of a rich research literature in many journals. This has resulted in the vast and flourishing development of this field, with the research community demonstrating significant growth and a constant increase in the scientific literature. As research in HIE continues to grow, it is essential to have a clearer understanding of its status, trends, and topics across the research field for researchers and participants.

This study explores the topic structure and evolutionary trends of HIE research by conducting a topic-based scientometric analysis of scientific articles concerning HIE from 2014 to 2023. Specifically, scientometric analysis is an effective way to evaluate research characteristics and frontiers in a specific research field, with the application of mathematical and statistical methodologies [11]. In particular, it can be employed to obtain a better understanding of what has been investigated in the past and further make predictions about what will happen in the future. Such analysis can depict the trends and distributions of the literature involving scientific impacts and productivity [12]. Meanwhile, topic modeling is a dimension reduction technology to extract hidden themes within a textual dataset, which detects term co-occurrence and assigns terms to the hidden themes [13]. It has been widely applied in scientific research trend analysis and in the identification of emerging topics within a particular research area [14,15]. Although scientometric and topic models have been popularly implemented in a variety of scientific fields, they have not been applied in the research field of HIE. Thus, this study contributes to deepening the understanding of HIE and provides valuable references and recommendations for researchers and potential contributors, focusing on the following questions:

1. What research topics were the HIE community interested in?
2. How did such research topics evolve over time?
3. Which countries/regions and institutions were the major contributors to HIE?
4. What were the scientific collaborations between countries/regions and institutions?
5. What were the main research concerns of prolific contributors?

## Methods

### Analysis Framework

**Figure 1:**
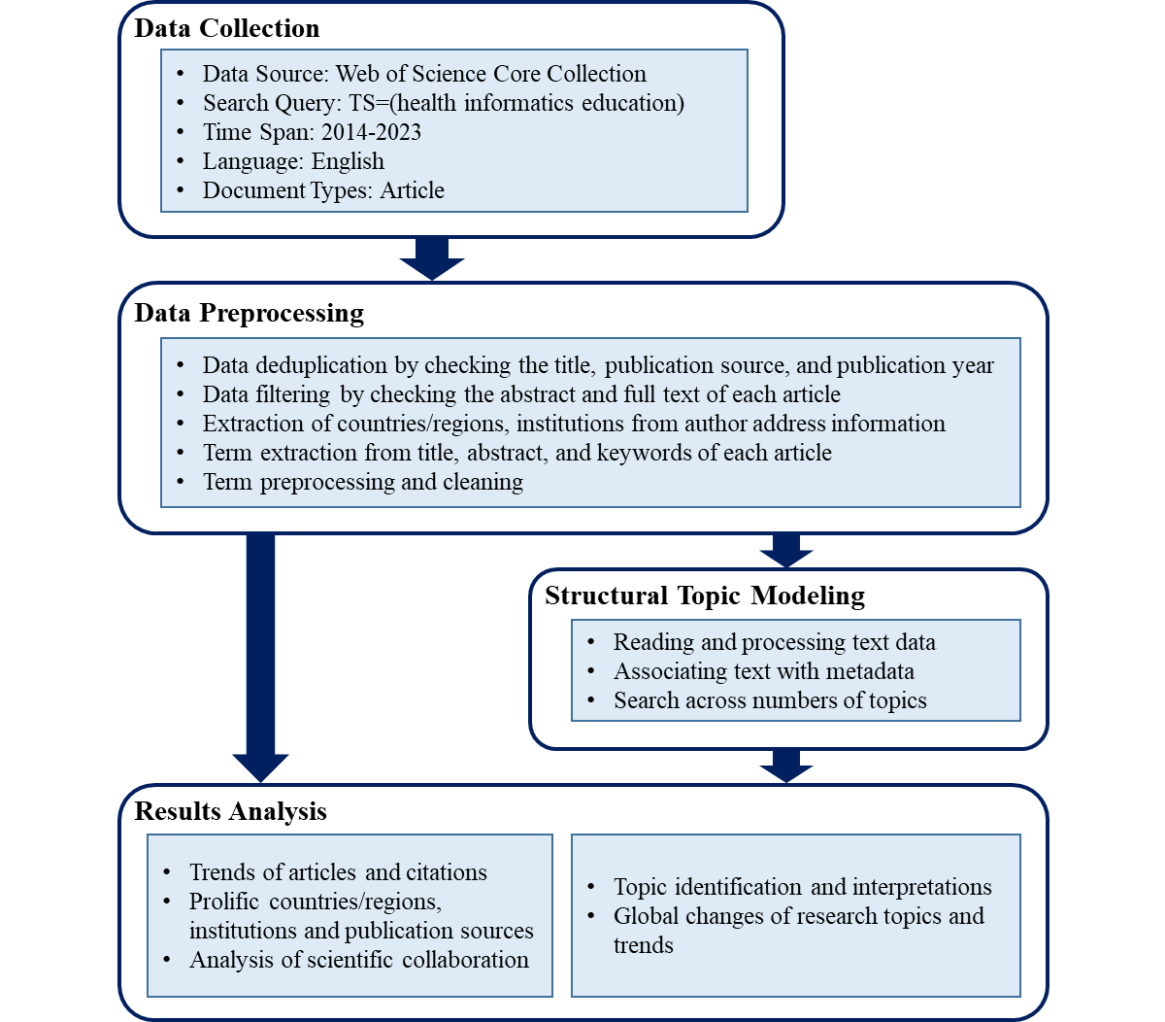
The scientometric analysis framework of health informatics education research.

The scientometric analysis framework of this paper is shown in Figure 1, which is mainly divided into 4 parts: data collection, data preprocessing, topic modeling, and results analysis.

### Data Collection

HIE-related scientific articles from 2014 to 2023 were retrieved from Web of Science Core Collection (WoSCC), using the search strategy “Topic=health informatics education, Year Published=2014-2023, Language=English, Document Type=Article.” The search was conducted using the following query: TS = (health informatics education) AND PY = (2014-2023) AND LA = (English) AND DT = (Article).

Only WoSCC was used to retrieve articles owing to its comprehensive collection of multidisciplinary academic journals, its higher quality than other databases, and its status as an authoritative source for citation information, making it a primary scientific database and the most preferred destination for researchers from all domains [16,17]. The search was restricted to this term and did not include other terms related to health informatics, such as medical informatics, nursing informatics, or consumer health informatics, as this search strategy has been shown to be effective for scientometric analysis of health informatics [18,19]. In addition, only research articles were included as they tend to make more original contributions when compared to other types of publications [20]. As a result, a total of 577 articles were retrieved.

It is commonly agreed that titles, abstracts, and article keywords are suitable for conceptual reviews because they usually represent the noteworthy content of articles [21,22]. In particular, abstracts are able to present summaries of articles in terms of research aims and problems, as well as major findings [23,24]. Therefore, in this study, the primary materials of topic modeling were the title, keywords, and abstract of each article. Of the articles collected, 2 without abstracts were excluded, and 575 articles with abstract information were selected for topic modeling.

### Data Preprocessing

To improve data quality before conducting topic modeling, preprocessing was performed, which involved the following 3 aspects.

First, the names of countries/regions, institutions, and researchers were extracted from the authors’ addresses, which were further preprocessed to ensure unified expressions. Terms were extracted from the titles, keywords, and abstracts, and were consolidated when they had multiple spellings.

Second, numbers, punctuations, symbols, and stop words (eg, “me,” “I,” “or,” “him,” “a,” and “they”) were deleted to enhance consistency and reduce computational load [25]. Punctuations were removed, and all the remaining terms were converted to lowercase.

Third, following the previous work [20], the weights 0.4, 0.4, and 0.2 were assigned to terms extracted from titles, keywords, and abstracts, respectively. Since in topic modeling, the estimations of document-topic and topic-term distributions are based on the document-term distribution, which is frequency-based, the assignment of weights was performed by multiplying the frequency by weight. For example, for each document, if *f*_1wi_, *f*_2wi_, and *f*_3wi_ denote the frequencies of the word *w_i_* in the keywords, title, and abstract, respectively, then the weighted frequency of the word *w_i_* in the document is denoted by 0.4×*f*_1wi_+0.4×*f*_2wi_+0.2×*f*_3wi_.

### Data Analysis

The major research topics within the 575 research articles in relation to HIE were uncovered by using the structural topic model (STM) [26]. The STM belongs to the class of topic models seeking to discover latent thematic clusters within a collection of texts, and is an extension of the latent Dirichlet allocation (LDA) model [27] and the correlated topic model (CTM) [28]. Traditional topic modeling techniques, such as LDA, assume that topics are discrete and independent. However, in many cases, topics may not be independent, and documents may have complex relationships between topics. The STM addresses these limitations by incorporating structural information into the topic modeling process. Besides identifying topics, the STM estimates the prevalence of the found topics by incorporating document-level covariates (eg, time of publication or number of authors).

**Figure 2:**
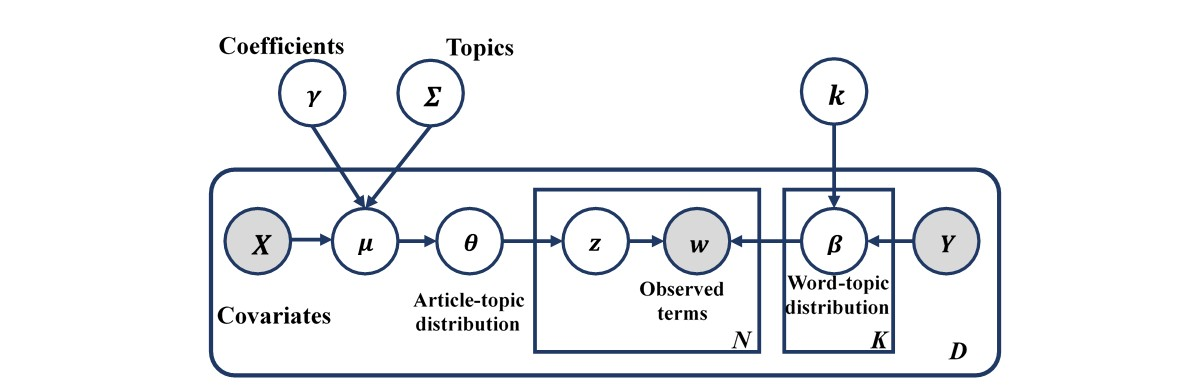
A diagram of the structural topic model.

A diagram of the STM, adapted from a recent study using the STM to analyze smart health care [29], is presented in Figure 2. The process of topic modeling is to estimate the *θ* and *β* based on the observed term *W*, which represent document topic and topic word distributions, respectively, where *n*∈{1,2,…,*N*} denotes terms covering a document, *k*∈{1,2,…,*K*} is the topic, *d*∈{1,2,…,*D*} represents the document number, and *z* represents the underlying topic allocation of each term.

During topic modeling, the decision of a value indicating the number of topics is a priority. Previous work has demonstrated that an exclusive reliance on statistical measures could result in a less meaningful model parameter choice [30]. Thus, following the suggestion of previous studies [31], 10 candidate models were adopted with the numbers of topics ranging from 4 to 15. Two domain experts, who have taught health informatics for more than 10 years, independently compared the outputs of the 10 models by evaluating the representative terms and articles to select the best-fitted model based on the following criteria adopted in previous work [32]: (1) Representative terms in each topic could form a meaningful topic together; (2) The top representative articles for each topic were in close relation to the topic; (3) There was limited overlap between topics within a topic model; and (4) All obvious research topics in HIE were included. In this study, both the experts chose the 7-topic model. The output of the model involved topic content and topic prevalence. The 7-topic model showed good semantic consistency within topics and exclusivity between topics.

Topics were labeled manually according to the inspection of the highest ranking 12 terms and 20 articles that consisted of each topic. The labels were individually examined by the 2 domain experts, who then reached an agreement through discussion. Specifically, they first examined the most representative terms for each topic and interpreted the discriminating terms within topics based on their semantic meanings. Then, they examined a sample of representative articles for each of the topics. Finally, they compared the labeling results and discussed the inconsistent labels to unify the results.

In addition to the STM, other methods and tools have also been adopted in this study. On the one hand, several scientometric indicators, namely, article count, citation count, and average citations per article, were used to evaluate the performance of involved publication sources, subjects, institutions, and countries/regions. On the other hand, scientific collaborations among countries/regions and institutions were explored by social network analysis using VOSviewer. VOSviewer generated collaborative networks by representing countries/regions and institutions as nodes, and the collaborative relationships were reflected using lines. The node size indicated the number of articles in which 2 linked countries/regions or institutions collaborated, and colors reflected the countries/regions or institutions.

## Results

### Annual Trends of Publications

**Figure 3:**
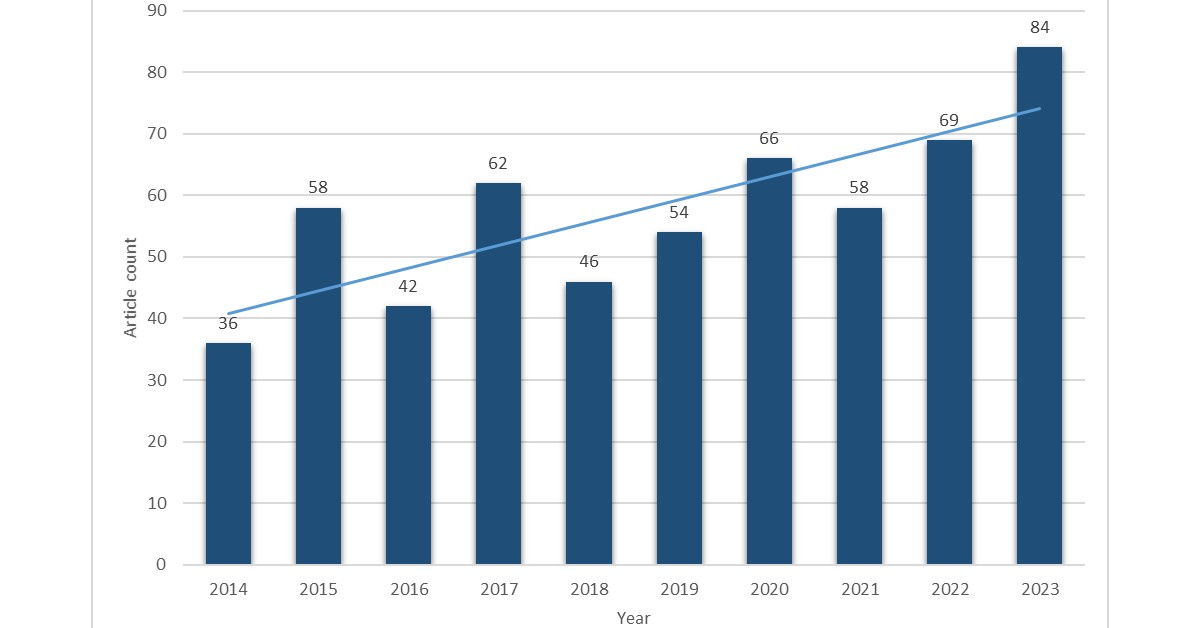
Number of articles published over time.

The annual trends of the article counts are depicted in Figure 3. Research in HIE has been growing during the studied period, particularly since 2020, reaching 84 articles in 2023.

Although there were fluctuations in the data, it is reasonable to conclude that HIE has received a growing interest from the academic community. Therefore, it is highly likely that the amount of HIE research will continue to increase in the near future.

### Prolific Countries/Regions and Institutions

In the analyses concerning countries/regions and institutions, all the countries/regions and institutions contributing to each article were included in the data, and the most prolific ones were evaluated using 3 scientometric indicators, namely, article count, citation count, and average citations per article.

A total of 70 countries/regions contributed to the 575 articles, and the top 10 countries/regions ranked by the article count are shown in Table 1. United States had 326 articles, followed by Canada (50 articles) and Australia (45 articles). These top 3 countries were also ranked among the top 3 in terms of citation count, with 4705, 673, and 514 citations, respectively.

Regarding average citations per article, the top 3 countries/regions included Germany (17.61), United States (14.43), and Canada (13.46). Although Germany had fewer articles (n=28) as compared to the top 3 countries, their articles had higher quality as indicated by the average citations per article. By considering all the abovementioned indicators, the performance of Germany is worth highlighting, indicating its great contribution to the research field of HIE.

The 575 articles concerning HIE were contributed by 200 institutions, among which the top 10 are shown in Table 2. Among the listed 10 institutions, 9 are from the United States, which again indicates the dominant position and active role of the United States in the field of HIE. Harvard University was the most prolific institution with 33 articles, followed by University of Texas System (28 articles) and University System of Ohio (24 articles).

Regarding citation count, the top 3 institutions were University of California System (521 citations), University of Minnesota System (394 citations), and Indiana University System (327 citations). These top 3 institutions were also ranked among the top 3 in terms of the average citations per article, with values of 26.05, 19.7, and 16.35, respectively.

By considering all these indicators, the performance of University of California System is worth highlighting, indicating its great contribution to the field of HIE. If we put Harvard University and Harvard Medical School together, we get an institution with the most influence regarding the indicators of article count and citation count.

**Table 1:** Top 10 countries/regions ranked by article count.

Country/region	Article count	Citation count (ranking)	Average citations per article (ranking)	Web of Science category (article count)
United States	326	4705 (1)	14.43 (2)	Medical Informatics (130) Health Care Sciences Services (115)
Canada	50	673 (2)	13.46 (3)	Medical Informatics (19) Health Care Sciences Services (14)
Australia	45	514 (3)	11.42 (6)	Medical Informatics (14) Health Care Sciences Services (13)
England	33	369 (5)	11.18 (7)	Medical Informatics (9) Health Care Sciences Services (9)
Germany	28	493 (4)	17.61 (1)	Medical Informatics (17) Health Care Sciences Services (15)
Netherlands	25	327 (6)	13.08 (4)	Medical Informatics (17) Health Care Sciences Services (15)
China	23	289 (7)	12.57 (5)	Medical Informatics (8) Health Care Sciences Services (4)
Brazil	20	168 (8)	8.40 (10)	Nursing (9) Medical Informatics (5)
Sweden	17	153 (9)	9.00 (9)	Health Care Sciences Services (9) Medical Informatics (8)
Spain	13	145 (10)	11.15 (8)	Medical Informatics (6) Health Care Sciences Services (6)

**Table 2:** Top 10 institutions ranked by article count.

Institution	Country	Article count	Citation count (ranking)	Average citations per article (ranking)	Web of Science category (article count)
Harvard University	United States	33	321 (4)	9.73 (9)	Health Care Sciences Services (21) Medical Informatics (15) Computer Science Information System (9)
University of Texas System	United States	28	274 (7)	9.79 (8)	Medical Informatics (15) Health Care Sciences Services (11) Computer Science Information System (7)
University System of Ohio	United States	24	317 (5)	13.21 (5)	Medical Informatics (7) Nursing (7) Computer Science Interdisciplinary Applications (5)
Harvard Medical School	United States	20	252 (8)	12.60 (6)	Health Care Sciences Services (12) Medical Informatics (6) Computer Science Information System (5)
Indiana University System	United States	20	327 (3)	16.35 (3)	Medical Informatics (11) Health Care Sciences Services (10) Computer Science Information System (7)
University of California System	United States	20	521 (1)	26.05 (1)	Health Care Sciences Services (12) Medical Informatics (11) Computer Science Interdisciplinary Applications (7)
University of Minnesota System	United States	20	394 (2)	19.70 (2)	Health Care Sciences Services (9) Medical Informatics (8) Health Policy Services (5)
University of Pennsylvania	United States	19	226 (9)	11.89 (7)	Medical Informatics (8) Health Care Sciences Services (6) Computer Science Information System (5)
State University System of Florida	United States	18	282 (6)	15.67 (4)	Medical Informatics (10) Health Care Sciences Services (8) Computer Science Interdisciplinary Applications (6)
University of Toronto	Canada	18	146 (10)	8.11 (10)	Medical Informatics (7) Health Care Sciences Services (6) Nursing (4)

### Top Publication Sources and Subjects

The 575 articles concerning HIE were contributed by 197 publication sources, among which the top 20 with a minimum article count of 5 are shown in Table 3, which together contributed to 51.3% (295/575) of the total studied articles. Journal of Medical Internet Research was the most prolific in the field with 42 articles, followed by Journal of the American Medical Informatics Association (37 articles), Applied Clinical Informatics (30 articles), and BMJ Open (28 articles). Regarding citation count, the top 3 journals were Journal of Medical Internet Research (1213 citations), Journal of the American Medical Informatics Association (442 citations), and Academic Medicine (328 citations). Regarding average citations per article, the top 3 journals were Academic Medicine (41.00), Journal of Medical Internet Research (28.88), and JMIR mHealth and uHealth (25.56). By considering all these indicators, the performance of Journal of Medical Internet Research is worth highlighting, indicating its significant contribution to the research field of HIE.

The 575 articles concerning HIE were distributed in 80 Web of Science categories, among which the top 20 with a minimum article count of 7 are shown in Table 4. Medical Informatics was the most relevant category in this field with 229 articles, followed by Health Care Sciences Services (189 articles) and Nursing (103 articles). The latter 2 categories also ranked among the top 2 from the perspective of citation count. Regarding average citations per article, the top 3 categories were Dentistry Oral Surgery Medicine (40.00), Education Scientific Disciplines (18.42), and Health Care Sciences Services (15.87). Although the article count of Dentistry Oral Surgery Medicine was only 7, its citation count was relatively high. That is because an article titled “Artificial Intelligence in Dentistry: Chances and Challenges” received widespread attention. This article reviewed artificial intelligence (AI) in dentistry and suggested that dental education needs to accompany the introduction of clinical AI solutions by fostering digital literacy in the future dental workforce.

**Table 3:** Top 20 publication sources ranked by article count.

Publication source	Article count	Citation count (ranking)	Average citations per article (ranking)	Web of Science category (article count)
Journal of Medical Internet Research	42	1213 (1)	28.88 (2)	Health Care Sciences Services (42) Medical Informatics (42)
Journal of The American Medical Informatics Association	37	442 (2)	11.95 (7)	Computer Science Information Systems (37) Computer Science Interdisciplinary Applications (37) Health Care Sciences Services (37) Information Science Library Science (37) Medical Informatics (37)
Applied Clinical Informatics	30	245 (5)	8.17 (12)	Medical Informatics (30)
BMJ Open	28	177 (8)	6.32 (14)	Medicine General Internal (28)
CIN: Computers Informatics Nursing	27	280 (4)	10.37 (10)	Computer Science Interdisciplinary Applications (27) Medical Informatics (27) Nursing (27)
International Journal of Medical Informatics	17	67 (14)	3.94 (17)	Computer Science Information Systems (17) Health Care Sciences Services (17) Medical Informatics (17)
BMC Medical Informatics and Decision Making	15	142 (9)	9.47 (11)	Medical Informatics (15)
Nurse Education Today	10	216 (7)	21.60 (4)	Education Scientific Disciplines (10) Nursing (10)
JMIR Medical Informatics	9	26 (17)	2.89 (19)	Medical Informatics (9)
JMIR mHealth and uHealth	9	230 (6)	25.56 (3)	Health Care Sciences Services (9) Medical Informatics (9)
Methods of Information in Medicine	9	98 (12)	10.89 (8)	Computer Science Information Systems (9) Health Care Sciences Services (9) Medical Informatics (9)
Academic Medicine	8	328 (3)	41.00 (1)	Education Scientific Disciplines (8) Health Care Sciences Services (8)
International Journal of Environmental Research and Public Health	8	84 (13)	10.50 (9)	Environmental Sciences (8) Public Environmental Occupational Health (8)
Journal of Interprofessional Care	8	62 (15)	7.75 (13)	Health Care Sciences Services (8) Health Policy Services (8)
BMC Medical Education	7	23 (19)	3.29 (18)	Education Educational Research (7) Education Scientific Disciplines (7)
Health Informatics Journal	7	40 (16)	5.71 (15)	Health Care Sciences Services (7) Medical Informatics (7)
Informatics for Health Social Care	7	104 (11)	14.86 (6)	Health Care Sciences Services (7) Medical Informatics (7)
Frontiers in Public Health	6	108 (10)	18.00 (5)	Public Environmental Occupational Health (6)
Journal of Nursing Education	6	24 (18)	4.00 (16)	Nursing (6)
Healthcare	5	13 (20)	2.60 (20)	Health Care Sciences Services (5) Health Policy Services (5)

**Table 4:** Top 20 Web of Science categories ranked by article count.

Web of Science category	Article count	Citation count (ranking)	Average citations per article (ranking)
Medical Informatics	229	371 (9)	1.62 (20)
Health Care Sciences Services	189	3000 (1)	15.87 (3)
Nursing	103	1326 (2)	12.87 (7)
Computer Science Interdisciplinary Applications	71	873 (3)	12.30 (8)
Computer Science Information Systems	70	697 (5)	9.96 (12)
Medicine General Internal	60	517 (8)	8.62 (14)
Public Environmental Occupational Health	46	593 (6)	12.89 (5)
Information Science Library Science	41	528 (7)	12.88 (6)
Education Scientific Disciplines	38	700 (4)	18.42 (2)
Health Policy Services	30	348 (10)	11.60 (15)
Pharmacology Pharmacy	14	221 (12)	15.79 (4)
Environmental Sciences	12	89 (15)	7.42 (16)
Medicine Research Experimental	10	95 (14)	9.50 (13)
Pediatrics	10	73 (18)	7.30 (17)
Radiology Nuclear Medicine Medical Imaging	10	121 (13)	12.10 (9)
Education Educational Research	8	26 (20)	3.25 (19)
Multidisciplinary Sciences	8	41 (19)	5.13 (18)
Oncology	8	88 (16)	11.00 (10)
Primary Health Care	8	85 (17)	10.63 (11)
Dentistry Oral Surgery Medicine	7	280 (11)	40.00 (1)

### Scientific Collaboration

**Figure 4:**
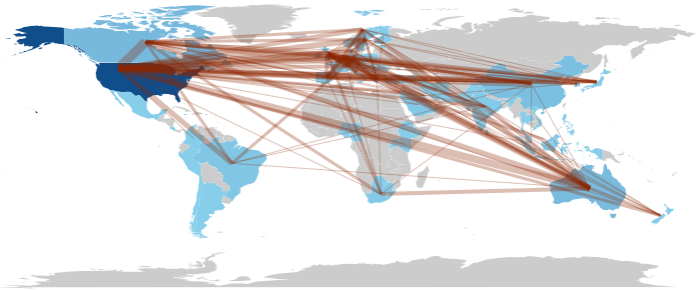
A macro-level picture of collaboration among countries (world map).

The macro picture of collaboration among countries in the research field of HIE from 2014 to 2023 is shown in Figure 4, indicating that related research in countries has benefited from international cooperation.

**Figure 5:**
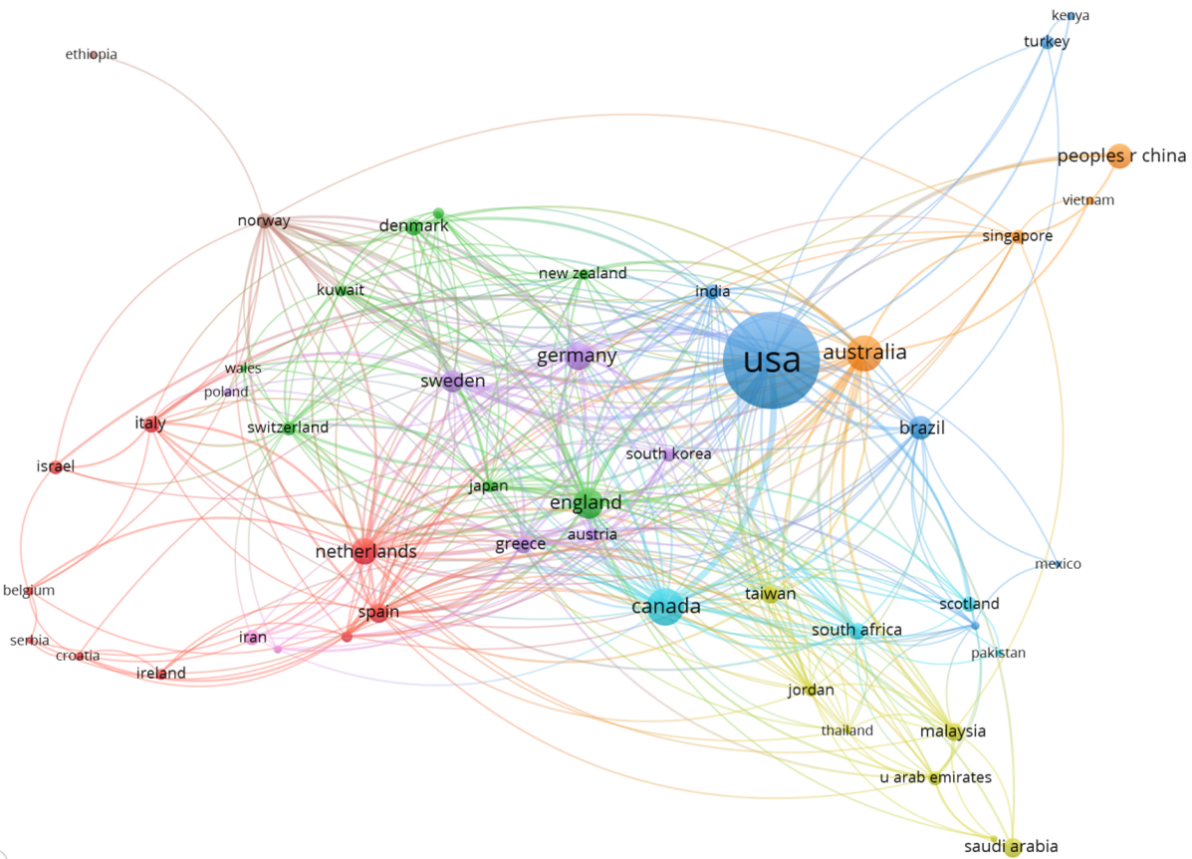
Collaboration network of countries/regions with a minimum article count of 2.

**Figure 6:**
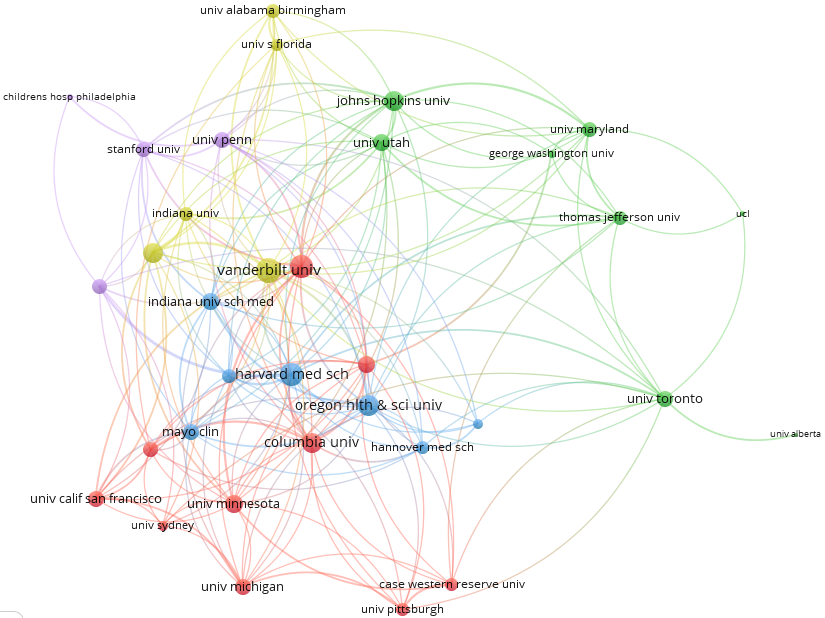
Collaboration network of institutions with a minimum article count of 2.

The collaborative scientific research networks among countries/regions and institutions are visualized in Figures 5 and 6, respectively. In these collaborative networks, countries/regions and institutions are represented by nodes, and collaborations between countries/regions or institutions are represented by edges. The size of each node indicates the number of articles from each country/region or institution, with a larger node being associated with a larger number of articles. Additionally, the color of a node is determined by the cluster to which the node belongs, and the lines between nodes represent collaborative relationships. The distance between 2 nodes in the visualization approximately indicates the relatedness of the countries/regions or institutions in terms of collaborative links. In general, the closer 2 countries/regions or institutions are located to each other, the stronger their relatedness.

For country/region collaborations, the minimum number of articles of a country/region was set to 2, and 50 countries met the threshold out of the 72 countries. For each of the 50 countries, the total strength of the co-authorship links with other countries was calculated. The results of the top 10 countries/regions are shown in Table 5. Of these 10 countries/regions, United States, England, and Canada collaborated with the most countries/regions, with 138, 85, and 82 collaborations, respectively.

For institution collaborations, the minimum number of articles of an institution was set to 2, and 36 institutions met the threshold out of the 1203 institutions. For each of the 36 institutions, the total strength of the co-authorship links with other institutions was calculated. The results of the top 10 institutions are shown in Table 6. Of these 10 institutions, Vanderbilt University, Harvard Medical School, and University of Washington collaborated with the most institutions, with 31, 28, and 27 collaborations, respectively.

**Table 5:** Top 10 countries/regions ranked by link strength.

Country/region	Total link strength	Article count
United States	138	326
England	85	33
Canada	82	50
Netherlands	76	25
Australia	75	45
Germany	67	28
Sweden	59	17
Greece	54	10
Norway	52	9
Spain	52	13

**Table 6:** Top 10 institutions ranked by link strength.

Institution	Total link strength	Article count
Vanderbilt University	31	18
Harvard Medical School	28	20
University of Washington	27	16
Oregon Health Science University	25	11
Columbia University	23	16
Johns Hopkins University	22	13
University of Texas Health Science Center Houston	22	13
University of Minnesota System	20	20
Indiana University System	18	20
University of Utah	18	14

### Major Research Topics and Trends

Table 7 shows the 7-topic STM results with representative terms under 4 different metrics, topic proportion within the whole corpus, and suggested topic labels. Among the 4 metrics, “Highest Prob” means terms with the highest probability, “FREX” weights words by their overall frequency and how exclusive they are to the topic, “Lift” weights words through division by their frequency in other topics, and “Score” divides the log frequency of the word in the topic by the log frequency of the word in other topics [26]. Topic proportion refers to the ratio of the number of articles on each topic to all articles. The results showed that Clinical Informatics Education (130/575, 22.6%) was the most researched topic among authors of HIE studies, followed by Mobile Application in Health Education & Healthcare (123/575, 21.4%) and Consumer Health Informatics Education (99/575, 17.2%). Additionally, a synopsis for each topic label has been provided to illustrate its meaning in the last column of Table 7.

**Figure 7:**
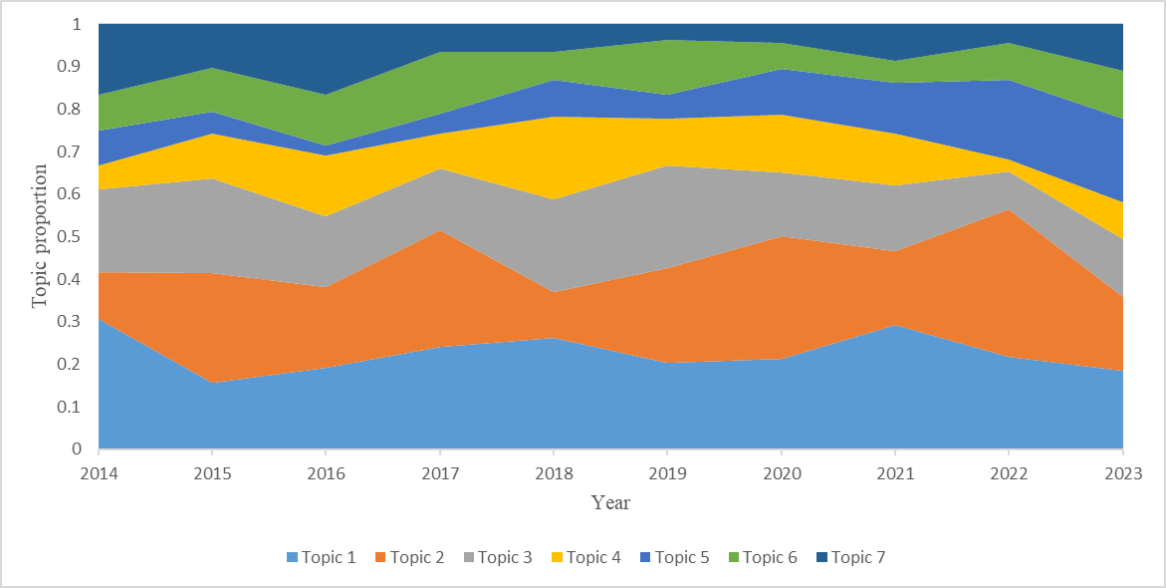
Annual topic proportion within the whole corpus for 7 topics.

Figure 7 shows the trends of the annual proportions of detected topics, which suggest that HIE research has become more diverse over time, with many different issues being of concern. The temporal dynamics of the distributions of all topics clearly indicate the unique foci for each year and demonstrate how the popularity of each topic has changed relative to other topics over time, depicting the process of development for health informatics research. For example, researchers showed continuous interest in Clinical Informatics Education (Topic 1) and Discipline of Biomedical and Health Informatics (Topic 6). In 2018, the predominant foci of the community were mainly related to Professional Teaching of Biomedical and Health Informatics (Topic 4). Years 2017 and 2022 witnessed a dramatic interest in issues concerning Mobile Application in Health Education & Healthcare (Topic 2). Some topics with high proportions at earlier time points showed reductions at the latest time point, such as Consumer Health Informatics Education (Topic 3) and Nursing Informatics Education (Topic 7). Since the year 2019, scholars in the field of HIE began to show interest in Public Health Informatics Education & Digital Health (Topic 5).

**Table 7:** The 7-topic structural topic model results with topic labels, representative terms under 3 different metrics, and topic proportions.

Topics	Topic labels	Representative terms^a^	Topic (N=575), n (%)	Synopsis of topic labels
Topic 1	Clinical Informatics Education	Highest Prob: care, clinical, system, record, provide, use, improve, health, medical, ehr, hospital, decision FREX: clinical, care, ehr, record, system, hospital, decision, provide, order, improve, set, plan Lift: ehr, clinical, decision, order, plan, care, record, hospital, system, efficient, team, point Score: ehr, clinical, record, care, hospital, system, decision, medical, order, provide, improve, train	130 (22.6)	Integrating clinical informatics into the full spectrum of medical education
Topic 2	Mobile Application in Health Education & Healthcare	Highest Prob: study, use, group, evaluate, education, assess, disease, application, result, mobile, factor, health FREX: mobile, disease, group, application, score, risk, evaluate, among, factor, age, random, assess Lift: mobile, random, score, lower, average, risk, disease, overall, mean, age, application, measure Score: mobile, disease, random, risk, score, age, factor, study, lower, group, application, test	123 (21.4)	Mobile apps are effective learning tools for medical education
Topic 3	Consumer Health Informatics Education	Highest Prob: health, information, use, online, access, social, user, study, engage, person, consumer, report FREX: social, online, user, access, information, consumer, person, engage, use, search, report, active Lift: consumer, social, user, online, person, access, engage, information, search, util, evid, source Score: consumer, information, health, user, social, online, access, person, use, engage, search, active	99 (17.2)	Health informatics from multiple consumer or patient perspectives, including health literacy and consumer education
Topic 4	Professional Teaching of Biomedical and Health Informatics	Highest Prob: health, education, competency, domain, skill, manage, requirement, public, need, new, core, advance FREX: domain, skill, requirement, competency, core, expert, advance, new, manage, gap, role, education Lift: domain, core, define, requirement, competency, skill, expert, advance, gap, employ, relevant, role Score: domain, competency, core, health, education, skill, public, expert, requirement, advance, new, define	61 (10.6)	Competencies of health informatics for health care professionals and others working in this field
Topic 5	Public Health Informatics Education & Digital Health	Highest Prob: health, medical, digit, education, learn, train, need, population, public, future, school, teach FREX: digit, learn, train, population, medical, teach, future, school, need, health, global, service Lift: digit, learn, teach, global, population, train, rapid, innovate, school, future, organ, achieve Score: digit, medical, learn, train, health, population, public, school, teach, need, education, active	56 (9.7)	Courses on digital health to train public health professionals
Topic 6	Discipline of Biomedical and Health Informatics	Highest Prob: data, science, medical, course, education, public, nation, integrate, year, include, model, intern FREX: science, data, course, nation, intern, field, year, computer, term, paper, center, topic Lift: science, course, data, intern, field, term, topic, nation, paper, computer, center, article Score: science, data, course, medical, public, school, nation, field, topic, intern, computer, integrate	55 (9.6)	Practices in teaching and learning in the discipline of biomedical and health informatics
Topic 7	Nursing Informatics Education	Highest Prob: nursing, competency, education, use, study, information, experience, record, integrate, assess, clinical, valid FREX: nursing, competency, experience, valid, purpose, prepare, integrate, innovate, scale, critical, record, study Lift: nursing, prepare, competency, scale, purpose, valid, innovate, experience, guide, unit, ability, critical Score: nursing, competency, record, information, study, innovate, clinical, integrate, academic, prepare, scale, experience	51 (8.9)	Digital competencies for nurses and nursing care in this digital age

^a^“Highest Prob” means terms with the highest probability, “FREX” weights words by their overall frequency and how exclusive they are to the topic, “Lift” weights words through division by their frequency in other topics, and “Score” divides the log frequency of the word in the topic by the log frequency of the word in other topics.

## Discussion

### Principal Findings

This study conducted a scientometric analysis on research of HIE from 2014 to 2023. Over the last decade, research on HIE has appeared to be increasing steadily. This field has been progressively developing and maturing, and has received a growing interest from the academic community. It is currently experiencing a phase of rapid growth.

The United States has made substantial contributions to the growth of publications in this research field. Additionally, Canada, Australia, England, Germany, China, Brazil, Sweden, and Spain demonstrated a high interest as well. Harvard University and Harvard Medical School represented the most influential institutions, and the most vigorous institutions included University of Texas System, University System of Ohio, Indiana University System, and University of California System. Research on HIE has been predominantly favored by journals related to health informatics, as indicated by article counts and citation counts. Journal of Medical Internet Research, Journal of The American Medical Informatics Association, and Applied Clinical Informatics were found to be the most influential and productive publication sources in terms of average citations per article and article counts. Collaboration among universities in the United States, England, and Canada was found to be high. Collaboration was identified to be mainly concentrated in the Americas, Europe, and Asia, with limited international collaboration from developing countries, suggesting that international collaboration in this research field needs to be strengthened.

Topic modeling analysis over time identified the knowledge structure and development trends in this research field. Seven topics were identified using R package STM, and representative terms were provided for each topic under 4 different metrics. Clinical Informatics Education was found to be the most researched topic among authors of HIE studies, followed by the topics of Mobile Application in Health Education & Healthcare and Consumer Health Informatics Education. Additionally, the temporal dynamics of the distributions of all topics have been presented, which demonstrated how the popularity of each topic has changed relative to other topics over time and thus depicted the process of development for health informatics research.

### Implications of the 7 Topics

For a profound understanding of the identified research topics of HIE, the most representative research works for each topic were analyzed.

#### Topic 1: Clinical Informatics Education

Education in clinical informatics is important for not only medical students but also other clinical staff at all professional levels of education. Integrating clinical informatics into the full spectrum of medical education is a vital step required for the understanding and practice of modern medicine. Education in clinical informatics is present throughout the spectrum of formal medical education, extending from high school to postgraduate training. Mai et al [33] presented a model of a clinical informatics curriculum that has been in place at the Children’s Hospital of Philadelphia. This educational model can support resident involvement in hospital-wide informatics efforts. Similar models to promote resident engagement in clinical informatics across training programs have the potential to accelerate health care improvements and develop the clinical informaticians of the future.

#### Topic 2: Mobile Application in Health Education & Healthcare

Current evidence suggests an important knowledge gain among health science students and professionals with mobile devices (m-learning). Matos Lamenha-Lins et al [34] conducted a study to verify whether a mobile app was a good learning tool for improving dental students’ knowledge. Mobile apps are effective learning tools that have a significant role in transferring information and knowledge to clinicians and nurses. The use of mobile technologies to improve health outcomes or mobile health is rapidly evolving. Garner et al [35] demonstrated that a mobile health app was effective in improving diabetes health literacy. Nezamdoust et al [36] performed a study to identify the factors affecting nurses’ use of practical health-related mobile apps in education and patient interaction based on the combined technology acceptance model (TAM) and diffusion of innovation (DOI).

#### Topic 3: Consumer Health Informatics Education

The American Medical Informatics Association defines consumer health informatics (CHI) as a field devoted to informatics from multiple consumers or patient views and which includes patient-focused informatics, health literacy, and consumer education [37]. Scholars mainly conducted research from 3 aspects, including consumer health needs [38], the effect of health education materials [39,40], and consumer health behavior and literacy [41,42].

#### Topic 4: Professional Teaching of Biomedical and Health Informatics

Health informatics has acquired the attributes of a profession, such as a well-defined set of competencies, a certification of fitness to practice, shared professional identity, life-long commitment, and a code of ethics. Research on this topic involves the following: health informatics curricula at different education levels [43,44], essential skills and competencies for health care professionals and others working in the field of health informatics to support the development and implementation of health informatics in health care [10,45,46], and the quality of teaching and training with delivered HIE programs [47-49].

#### Topic 5: Public Health Informatics Education & Digital Health Education

A generally accepted definition of public health informatics is as follows: “the systematic application of information, computer science, and technology to public health practice, research, and learning” [50]. Public health has a strong historical basis in handling health information, and much of public health activity continues to involve collecting, analyzing, and disseminating information about the health of the public. Moreover, public health is inherently interdisciplinary, and the sciences underlying public health encompass a set of skills and knowledge that are recognized to be at the core of health informatics [51]. Meanwhile, digital health has been recognized as an innovative health solution that enables public health action to stop the rapid spread of a virus during a pandemic [52]. Digital health is a key driver in addressing public health challenges and achieving sustainable health systems and universal health coverage [53]. Therefore, there is an urgent need to analyze how to establish systematic courses on digital health in medical schools to train the next generation of doctors and public health managers in the integration of health theory and digital technology [54].

#### Topic 6: Discipline of Biomedical and Health Informatics

Health informatics became a professional discipline nearly 2 decades ago [55]. Being an interdisciplinary discipline according to its definition, health informatics draws upon different disciplines. Professionals entering the field have diverse backgrounds, and HIE at various levels needs to support a range of work roles. To help inform programs on practices in teaching and learning, a survey of master’s programs in health and biomedical informatics in the United States was conducted to determine the national landscape of culminating experiences, including capstone projects, research theses, internships, and practicums [56]. Bellazzi et al [57] provided a description of the fundamental design principles of 3 medical informatics education programs and explored some aspects of the teaching modules, highlighting their positive aspects.

#### Topic 7: Nursing Informatics Education

Nursing informatics is a subcomponent of health informatics pertaining to nurses and nursing care [58]. Nurses need digital competencies to integrate new technologies in their professional activities. The foundations for these digital competencies are acquired during their vocational training/undergraduate education and ongoing training, and are deepened in their workplaces [59]. Most research involving this topic was focused on improving competencies for nursing in the digital age [60,61].

### Comparison With the Literature

As IT is applied widely in health care and medical education, some studies have examined the evolution of the intellectual and conceptual structure in the field of HIE. For example, Monkman et al [62] conducted a scoping review to identify health informatics competencies from research articles and grey literature originating from professional associations, and proposed a health informatics core competencies framework that could be used to inform Canadian health informatics programs to ensure graduates would be prepared for careers in health informatics. To outline the history of medical informatics education in China, Hu et al [63] systematically analyzed the status of medical informatics education at different academic levels and suggested reasonable strategies for the further development of the field in China. Asiri [64] explored the challenges that face HIE and training in the Kingdom of Saudi Arabia by examining the state of health IT education, with a focus on HIE. Jeon et al [65] presented the status of nursing informatics education and the content covered in nursing informatics courses in Korea, and concluded that a greater focus was needed on training faculty and developing the courses. Similarly, Masic et al [66] investigated the status of medical informatics education in Bosnia and Herzegovina, and Europe.

Although these studies made invaluable contributions to the understanding of HIE, they tended to focus on specific regions or disciplines and did not provide a global overview of research in HIE. Additionally, they did not use any topic modeling methods to reveal topic structure and evolutionary trends of this research field, and thus, the methodologies employed in these studies had some limitations. Meanwhile, there was a scarcity of comprehensive scientometric analysis in this research field. Therefore, this study provided a topic modeling–based scientometric analysis of scientific articles concerning HIE, and it analyzed aspects such as productive countries and institutions, scientific collaborations, and topics and trends of HIE research.

### Limitations

The main limitation of this study was that only 1 database was searched to identify studies for inclusion, meaning that potentially relevant studies could have been overlooked. Although WoSCC is a widely used academic database globally, other medical-oriented databases may provide more significant academic and practical information. Subsequent studies should conduct searches in commonly used databases, such as PubMed, Scopus, IEEE, and Google Scholar, to ensure a more comprehensive analysis. Another constraint was that only journal articles were retrieved for analysis, excluding other relevant publication types in this field, such as reviews, book chapters, and conference proceedings. This could potentially affect the summarization and generalization of the results.

As AI has covered most of the research topics in health informatics, academic research on the intersection of AI and HIE has emerged in recent years. For example, Paranjape et al [67] summarized the state of medical education and recommended a framework to include AI education. Yang et al [68] examined the impact of an AI system tutoring course on clinical training and found an increased improvement in the expert-led AI tutoring group. However, AI did not appear as a representative term in any of these topics. The main reason may be that the time span chosen for this study was long at 10 years, and AI did not dominate during this period. Overall, the application of AI in HIE should be taken seriously, and formal training for clinical and health informatics programs should consist of AI technology and its applications.

### Conclusion

The field of HIE is increasingly promising and active, with academic products flourishing. This study presents the results and findings from a topic-driven scientometric analysis of academic research articles concerning HIE published from 2014 to 2023. This study not only highlights the major research concerns and issues among scholars but also identifies the tendencies of article and citation counts; identifies major countries/regions, institutions, publication sources, and subjects; and outlines academic collaboration relationships. The topic-based scientometric analysis contributed to HIE by providing a comprehensive overview of its community. The contributions of this study with regard to HIE have been summarized.

First, it helps researchers, policymakers, and practitioners better understand the historical, extant, and upcoming structure of research in HIE. The exploration of major topics and their trends is helpful for the identification and comparison of current and potential scientific strength. Research governors or funding agencies can optimize research policies to promote potential competitive research areas and enhance scientific collaborations in specific research areas in order to bolster scientific and educational activities. Second, findings regarding prolific countries/regions and institutions can assist scholars in sharing research, conducting collaborations, and identifying important actors from whom to learn. This is valuable for the research community of HIE in terms of understanding its research status and trends, especially by translating the findings into directions for future research. Third, this study provides a clear outline of HIE, which could help interested educators as well as researchers who wish to continue working in this area. The results of prolific publication sources and subjects provide suggestions about sources to which scholars can submit their work and will help scholars to better position their research in the field.

As it explores the structures and research topics within the area of HIE, this study serves as a starting point for further in-depth research into HIE. The research scope of HIE is relatively broad, and this study is expected to provide editors and conference organizers with ways to identify gaps in the field, leading them to create special issues or conference sessions about topics that are generally not co-occurring, which could create potential new synergies in HIE.

## Abbreviations

AI: artificial intelligence
HIE: health informatics education
LDA: latent Dirichlet allocation
STM: structural topic model
WoSCC: Web of Science Core Collection

## Data Availability

The data and analysis code are available upon reasonable request.
